# Type-1 metabotropic glutamate receptor signaling in cerebellar Purkinje cells in health and disease

**DOI:** 10.12688/f1000research.10485.1

**Published:** 2017-04-04

**Authors:** Masanobu Kano, Takaki Watanabe

**Affiliations:** 1 Department of Neurophysiology, Graduate School of Medicine, The University of Tokyo, Bunkyo-ku, Tokyo, Japan

**Keywords:** Purkinje cell, signaling, metabotropic glutamate receptor, cerebellum, cognitive function

## Abstract

The cerebellum is a brain structure involved in coordination, control, and learning of movements, as well as certain aspects of cognitive function. Purkinje cells are the sole output neurons from the cerebellar cortex and therefore play crucial roles in the overall function of the cerebellum. The type-1 metabotropic glutamate receptor (mGluR1) is a key “hub” molecule that is critically involved in the regulation of synaptic wiring, excitability, synaptic response, and synaptic plasticity of Purkinje cells. In this review, we aim to highlight how mGluR1 controls these events in Purkinje cells. We also describe emerging evidence that altered mGluR1 signaling in Purkinje cells underlies cerebellar dysfunctions in several clinically relevant mouse models of human ataxias.

## Introduction

**Figure 1.**
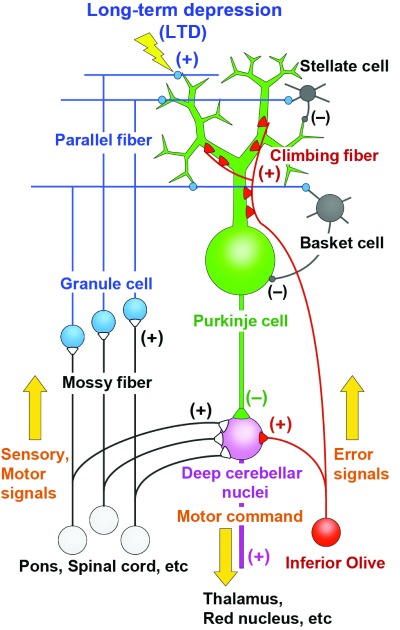
Simplified scheme of cerebellar neural circuitry.

The cerebellum is involved in coordination, control, and learning of movements and also in some aspects of cognitive functions ^1, 2^. Purkinje cells are the sole output neurons from the cerebellar cortex and receive two distinct excitatory inputs, namely parallel fibers and climbing fibers ^1, 3, 4^ ( Figure 1). Parallel fibers are the axons of granule cells in the cerebellar cortex and form synapses on spines of Purkinje cell dendrites. Synaptic inputs from individual parallel fibers are weak, but numerous (as many as 100,000 in mice) parallel fibers innervate each Purkinje cell. Granule cells are driven by excitatory inputs from mossy fibers originating from various precerebellar nuclei and the spinal cord ( Figure 1). Mossy fibers are thought to convey sensory information arising from various body parts and motor command signals from the upper centers through mossy fibers ( Figure 1). Climbing fibers originate from the inferior olive in the contralateral medulla oblongata and form direct synaptic contacts with Purkinje cells ( Figure 1). A single Purkinje cell is innervated by only one climbing fiber in the adult cerebellum, but each climbing fiber makes hundreds of strong connections with Purkinje cell proximal dendrites ( Figure 1). Purkinje cells then form inhibitory synaptic connections on neurons in the deep cerebellar nuclei and vestibular nuclei ( Figure 1). Climbing fibers are thought to convey error signals that represent the mismatch between the motor command and the actual movement ^1, 5^ ( Figure 1). A predominant theory of cerebellar motor learning is based on long-term depression (LTD) that occurs at parallel fiber–Purkinje cell synapses when they are activated conjunctively with climbing fiber input for a certain period of time ^1, 5, 6^. Parallel fiber inputs associated with movement error will be depressed by LTD, and information flow through the cerebellar circuitry changes so as to support and facilitate the correct movements ^1, 5, 6^ ( Figure 1). Synaptic connections onto Purkinje cells from climbing fiber and parallel fibers as well as from inhibitory interneurons are formed and established during postnatal development ^7– 14^. Therefore, activity and synaptic responses of Purkinje cells, LTD at parallel fiber–Purkinje cell synapses, and establishment of synaptic wiring onto Purkinje cells during postnatal development are crucial factors for proper functions of the cerebellum.

The metabotropic glutamate receptor (mGluR) family of G-protein-coupled receptors (GPCRs) consists of eight members, mGluR1 to mGluR8, which are divided into group I (mGluR1 and mGluR5), group II (mGluR2 and mGluR3), and group III (mGluR4, mGluR6, mGluR7, and mGluR8) ^15, 16^. Group I mGluRs are coupled to the G-protein G _q_ family (G _q_ and G _11_), which mediates inositol trisphosphate (IP _3_)-induced Ca ^2+^ mobilization and activation of protein kinase C (PKC). Purkinje cells in the cerebellum strongly express mGluR1 ^17– 20^, and many studies have shown that mGluR1 signaling is essential for various aspects of cerebellar function ^21– 24^. A crucial role for mGluR1 in Purkinje cells is best illustrated by the multiple phenotypes of cerebellar dysfunction in mGluR1-knockout mice ^25– 27^, which can be rescued by Purkinje cell-specific re-expression of mGluR1 ^28, 29^. Moreover, dysregulation of mGluR1 signaling in Purkinje cells has been found in several clinically relevant mouse models of human cerebellar ataxias, and mutations of mGluR1 and related molecules have been reported in certain types of human ataxias ^24, 30^. Thus, this review aims to summarize the roles of Purkinje cell mGluR1 signaling in normal cerebellar functions and their dysfunctions relevant to human ataxias.

## Background

**Figure 2.**
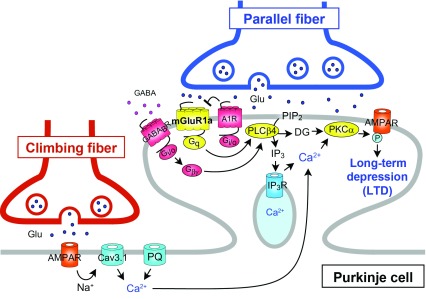
Schematic diagram of type-1 metabotropic glutamate receptor (mGluR1)-mediated long-term depression (LTD) at parallel fiber to Purkinje cell synapse. The long C-terminal domain of mGluR1a is required for inositol trisphosphate (IP _3_)-mediated Ca ^2+^ release and LTD induction. mGluR1b lacking this C-terminal domain cannot drive the cascade for LTD induction. A1R, A _1_-subtype adenosine receptor; AMPAR, α-amino-3-hydroxy-5-methyl-4-isoxazolepropionic acid receptor; DG, diacylglycerol; CaV3.1, Ca _V_3.1 T-type voltage-dependent Ca ^2+^ channel; GABA _B_R, type-B γ-aminobutyric acid receptor; PIP _2_, phosphatidylinositol 4,5-bisphosphate; PKC, protein kinase C; PLC, phospholipase C; PQ, P/Q-type voltage-dependent Ca ^2+^ channel.

**Figure 3.**
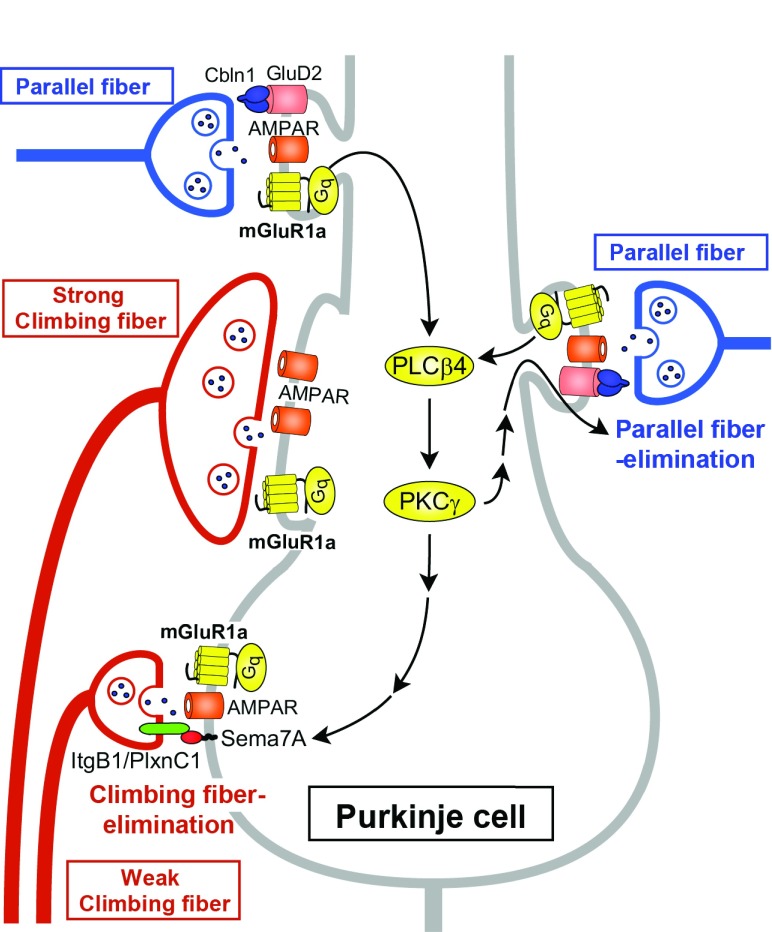
Schematic diagram of type-1 metabotropic glutamate receptor (mGluR1) signaling in Purkinje cells required for developmental synapse elimination. Parallel fiber synaptic inputs activate mGluR1 and its downstream signaling (Gq – phospholipase Cβ4 [PLCβ4] – protein kinase Cγ [PKCγ]) in Purkinje cells. Sema7A retrogradely acts on its Plexin C1 (PlxnC1)/Integrin B1 (ItgB1) receptor on “weak” climbing fibers and eliminates them from the soma during postnatal day (P)15 to P18. The same mGluR1 to PKCγ signaling eliminates parallel fiber synapses from proximal dendrites during P15 to P30. The long C-terminal domain of mGluR1a is required for climbing fiber synapse elimination. GluD2, glutamate receptor δ2.

Among a number of signaling molecules that have been identified to be involved in cerebellar LTD, mGluR1 and its downstream molecules constitute a canonical pathway for LTD (see Figure 2). Mutant mice lacking mGluR1 or its downstream molecules show deficient LTD, clear impairment of cerebellum-dependent motor learning, and motor discoordination ^25, 26^, supporting the notion that cerebellar LTD is a cellular basis of motor learning ^1, 5, 31^. It has also been shown that the mGluR1 signaling cascade is crucial for the elimination of redundant climbing fiber to Purkinje cell connections during postnatal cerebellar development ^27, 32^ (see Figure 3). This phenomenon is known to be a representative model of “synapse elimination” or “axon pruning” in the developing brain ^7, 8, 33– 36^. In mutant mice lacking mGluR1 or its downstream molecules (Gαq, phospholipase Cβ4, or PKCγ), multiple climbing fiber innervation of Purkinje cells persists into adulthood because of the impairment of climbing fiber elimination during the third postnatal week ^27, 32, 37– 39^ ( Figure 3). Importantly, deficient LTD, impaired motor learning, motor discoordination, and impaired climbing fiber synapse elimination are all restored by Purkinje cell-specific expression of mGluR1a, a predominant splice variant in Purkinje cells, into global mGluR1-knockout mice ^28^. This result clearly indicates that the mGluR1 cascade within Purkinje cells is essential for neural circuit development, synaptic plasticity, and motor learning in the cerebellum ^23, 40^.

**Figure 4.**
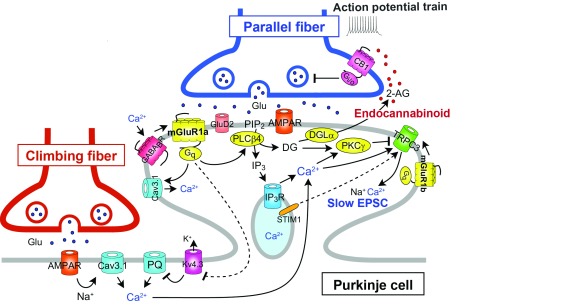
Schematic diagram showing type-1 metabotropic glutamate receptor (mGluR1)-mediated Ca ^2+^ mobilization and interaction between mGluR1 and other G-protein-coupled receptors (GPCRs) or ion channels. The long C-terminal domain of mGluR1a is required for proper perisynaptic localization of mGluR1 and inositol trisphosphate (IP _3_)-mediated Ca ^2+^ release but is dispensable for short transient receptor potential channel 3 (TRPC3)-mediated inward currents. 2-AG, 2-arachidonoylglycerol; CB1, cannabinoid receptor type-1; DGL, diacylglycerol lipase; EPSC, excitatory postsynaptic current; Kv4.3, Kv4.3 voltage-dependent K ^+^ channel; STIM1, stromal interaction molecule 1.

Activation of mGluR1 by repetitive parallel fiber stimulation induces slow excitatory postsynaptic potentials (EPSPs)/excitatory postsynaptic currents (EPSCs) in Purkinje cells ^41– 43^. This slow EPSC has been shown to be mediated by an inward cation current through the TRPC3 channel ^44^ ( Figure 4). Repetitive parallel fiber stimulation also induces mGluR1-mediated production of IP _3_ and local Ca ^2+^ release from internal stores in Purkinje cell dendrites ^45, 46^ ( Figure 4). Furthermore, the activation of mGluR1 by repetitive parallel fiber stimulation induces the release of an endocannabinoid that acts retrogradely on cannabinoid CB _1_ receptors on parallel fibers and climbing fibers and causes transient suppression of glutamate release from parallel fibers and climbing fibers ^47– 50^ ( Figure 4). The endocannabinoid that mediates retrograde synaptic suppression has been identified as 2-arachidonoylglycerol (2-AG), which is produced by diacylglycerol lipase α ^51, 52^. This retrograde signaling mediated by 2-AG is required for the induction of LTD at parallel fiber synapses ^53, 54^, and CB _1_ knockout mice exhibit a clear impairment of delay eyeblink conditioning, a representative of cerebellum-dependent motor learning ^55^.

Several studies have shown that mGluR1 interacts with another GPCR or ion channel either directly or through G-protein (see Figure 4). The mGluR1-mediated responses of Purkinje cells in cerebellar slices are enhanced by the activation of type-B γ-aminobutyric acid receptor (GABA _B_R) through G _i/o_ protein ^56^ ( Figure 4). In cultured Purkinje cells, GABA _B_R activation enhances LTD by elevating mGluR1 signaling via phospholipase Cβ activation by the G-protein β/γ subunit released as a consequence of GABA _B_R activation ^57^ ( Figure 2). Moreover, the activation of GABA _B_R enhances the mGluR1-mediated responses in cultured Purkinje cells through G _i/o_ protein-independent direct interaction between GABA _B_R and mGluR1 ^58^ ( Figure 4). Interestingly, this GABA _B_R–mGluR1 interaction does not require GABA but is caused by extracellular Ca ^2+^ ^58^ ( Figure 4). The dynamic range of mGluR1 is positively controlled by extracellular Ca ^2+^ such that sensitivity of mGluR1 to its agonist is enhanced at low dose range ^59^. This mGluR1 sensitization is absent in Purkinje cells lacking GABA _B_R, indicating that GABA _B_R can act as a Ca ^2+^-dependent cofactor of mGluR1 signaling in Purkinje cells ^58^ ( Figure 4). In contrast, mGluR1-induced inward current in Purkinje cells has been reported to be continuously depressed by activation of G _i/o_ protein-coupled A _1_-subtype adenosine receptor (A1R) ^60^ ( Figure 1). This inhibitory effect from A1R to mGluR1 was independent of G _i/o_ protein, suggesting a direct interaction between the two receptors. It has been shown that mGluR1-induced mobilization of Ca ^2+^ leads to activation of outward K ^+^ current that slowly hyperpolarizes Purkinje cells ^61, 62^. On the other hand, activation of mGluR1 potentiates Ca _V_3.1 T-type Ca ^2+^ channel currents in Purkinje cell dendritic spines through a G-protein- and tyrosine phosphatase-dependent mechanism ^63^ ( Figure 4).

Taken together, these studies clearly indicate that mGluR1 plays multiple and crucial roles in the regulation of synaptic wiring, excitability, synaptic transmission, and synaptic plasticity in Purkinje cells.

## New insight into the roles of mGluR1 in Purkinje cell function

### Synaptic plasticity and developmental synapse elimination in Purkinje cells

Ohtani *et al.* reintroduced mGluR1b, a short variant that lacks a long carboxyl-terminal domain, into Purkinje cells of global mGluR1-knockout mice (mGluR1b-rescue mice) ^29^. As mentioned above, restoration of mGluR1a, which contains the long carboxyl-terminal domain, rescued all the cerebellar deficits in mGluR1-knockout mice ^28^. In contrast, mGluR1b-rescue mice exhibited normal TRPC3-mediated slow EPSC and motor coordination but showed impairments in IP _3_-mediated Ca ^2+^ release, developmental climbing fiber synapse elimination, LTD at parallel fiber to Purkinje cell synapses, and delayed eyeblink conditioning ^29^. Furthermore, in mGluR1b-rescue mice, mGluR1b showed dispersed perisynaptic localization at Purkinje cell spines ^29^. This study indicates that the long C-terminal domain of mGluR1a is required for proper perisynaptic localization of mGluR1, IP _3_-mediated Ca ^2+^ release, developmental climbing fiber synapse elimination, LTD induction, and motor learning ( Figure 2– Figure 4). Chae *et al.* reported that blockade of TRPC3 channels by a broad-spectrum TRPC antagonist or by a TRPC3 antibody suppressed LTD induction at parallel fiber to Purkinje cell synapses ^64^. However, the dissociation between TRPC3-mediated slow EPSC and LTD in mGluR1b-rescue mice suggests that TRPC3-mediated slow EPSC is necessary but not sufficient for LTD induction.

As for developmental synapse elimination, Uesaka *et al.* demonstrated that Sema7A, a membrane-bound class of semaphorin, functions as a retrograde signaling molecule from Purkinje cells to losing climbing fibers at the downstream of mGluR1 ^65^ ( Figure 3). When Sema7A was knocked down in Purkinje cells by lentivirus-mediated RNA interference during postnatal development, climbing fiber synapse elimination was impaired from postnatal day 15 (P15). Double knockdown of Sema7A and mGluR1 in Purkinje cells caused impairment of climbing fiber synapse elimination to the same extent as single mGluR1 knockdown. Furthermore, expression of Sema7A was significantly reduced in the cerebellum of mGluR1-knockout mice. Importantly, overexpression of Sema7A in mGluR1-knockdown Purkinje cells restored normal climbing fiber synapse elimination. These data indicate that Sema7A mediates climbing fiber synapse elimination downstream of mGluR1 ^65^.

Ichikawa *et al.* revealed that massive elimination of parallel fiber synapses occurs from around P15 to P30, which requires mGluR1 signaling in Purkinje cells ^66^ ( Figure 3). Climbing fibers and parallel fibers innervate proximal and distal portions of Purkinje cell dendrites, respectively. In between, there is an intermediate dendritic portion with overlapped innervation by climbing fibers and parallel fibers. Ichikawa *et al.* showed that climbing fiber and parallel fiber territories expanded with marked enlargement of the regions of overlapping innervation until P15 ^66^. Then, the territories became segregated from P15 to around P30 by massive elimination of parallel fiber synapses from proximal dendrites ^66^. Interestingly, this parallel fiber synapse elimination was absent in mGluR1-knockout mice and also in PKCγ-knockout mice, and the defect of mGluR1-knockout mice was rescued by lentivirus-mediated expression of mGluR1a in mGluR1-deficient Purkinje cells ^66^ ( Figure 3). These findings give a new insight into roles of mGluR1 signaling in Purkinje cell synaptic wiring during postnatal development. mGluR1 signaling is essential for eliminating weaker climbing fiber synapses from the soma to establish mono-climbing fiber innervation and also for eliminating parallel fiber synapses from proximal dendrites to segregate climbing fiber and parallel fiber territories in Purkinje cell dendrites.

### Interaction of mGluR1 and another GPCR or ion channel

Kamikubo *et al.* explored the physiological relevance of direct interaction between A1R and mGluR1 ^67^. They first demonstrated that the two GPCRs closely co-localized and formed heteromeric complexes on the cell surfaces by using Förster resonance energy transfer analyses in cultured Purkinje cells ^67^ ( Figure 4). Then they showed evidence that A1R antagonizes the induction of LTD by decreasing the ligand sensitivity of mGluR1 but not the coupling efficacy from mGluR1 to the intracellular signaling cascades ^67^ ( Figure 2).

Otsu *et al.* showed that mGluR1 activity and Purkinje cell depolarization control climbing fiber-induced Ca ^2+^ influx ^68^. Under basal conditions, climbing fiber stimulation evoked Ca ^2+^ transients mainly in the proximal dendrites through T-type Ca ^2+^ channels. Combined mGluR1 activation and depolarization unlocked dendritic Ca ^2+^ spikes mediated by P/Q-type Ca ^2+^ channels through inactivation of the A-type K ^+^ channels in the distal dendrites of Purkinje cells ^68^ ( Figure 4). These results suggest that climbing fiber-induced Ca ^2+^ transients can be graded at parallel fiber synapses depending on their activity (i.e. the extent of mGluR1 activation) and therefore give new insight into the mechanisms of LTD induction at parallel fiber synapses.

Hartmann *et al.* demonstrated that both TRPC3-mediated slow EPSC and IP _3_-mediated Ca ^2+^ release following mGluR1 activation by repetitive parallel fiber stimulation were strongly attenuated in Purkinje cells lacking stromal interaction molecule 1 (STIM1) ^69^ ( Figure 4). In Purkinje cell-specific STIM1-knockout mice, both of the mGluR1-mediated responses were deficient and intracellular Ca ^2+^ stores were empty ^69^. Depolarization of STIM1-deficient Purkinje cells induced normal Ca ^2+^ entry through voltage-gated Ca ^2+^ channels, which restored TRPC3-mediated slow EPSC and IP _3_-mediated Ca ^2+^ release only transiently ^69^. Their results indicate that STIM1 is essential for the maintenance of normal Ca ^2+^ levels in the endoplasmic reticulum at rest and that TRPC3 activation is dependent on intracellular Ca ^2+^ level and requires interaction with STIM1 ( Figure 4).

Kato *et al.* reported that glutamate receptor δ2 (GluRδ2 or GluD2), PKCγ, and TRPC3 are major interactors of mGluR1 by an unbiased proteomic approach ^70^. They found that mGluR1-evoked inward currents were increased in a spontaneous mutant mouse line lacking GluD2, which disrupted the time course of mGluR1-dependent synaptic transmission at parallel fiber–Purkinje cell synapses. These results suggest that GluD2 is part of the mGluR1 signaling complex in Purkinje cells. In marked contrast, Ady *et al.* reported that mGluR1-mediated inward currents induced by repetitive parallel fiber stimulation were markedly reduced in Purkinje cells of another strain of spontaneous GluD2-deficient mice ^71^. They also showed that pharmacological blockade and genetic mutation of GluD2 channel pore reduced mGluR1-mediated slow EPSCs and claimed that inward currents through GluD2 channel constituted a significant portion of mGluR1-mediated slow EPSCs ^71^. Further studies are necessary to clarify whether and how mGluR1 and GluD2 interact to evoke physiologically relevant responses in Purkinje cells.

## Dysregulation of mGluR1 signaling in Purkinje cells in cerebellar diseases

### mGluR1 loss of function in Purkinje cells and cerebellar ataxia

Given that genetic deletion of mGluR1 signaling molecules in Purkinje cells causes clear ataxia in mice, many studies have been performed regarding dysregulation of mGluR1 signaling in mouse models of human cerebellar diseases, especially spinocerebellar ataxias (SCAs). In several mouse models of autosomal-dominant SCAs, the expansion of the CAG (Q) trinucleotide repeat disturbs transcription programs in the nucleus. Two types of SCA1 mouse models, SCA1 82Q and SCA1 154Q that express 82 and 154 Q repeats in the human ataxin-1 gene, respectively, have been generated ^72, 73^. In these SCA1 mouse models, loss of retinoid-related orphan receptor alpha (RORα)-mediated signaling leads to the reduced expression of mGluR1, TRPC3, and EAAT4, an excitatory amino acid transporter subtype specific to Purkinje cells ^74– 77^ ( Table 1). Furthermore, in the spontaneous ataxic mutant mouse *staggerer*, which exhibits mutation of the *RORα* gene and therefore is similar to SCA1 mouse models, mGluR1 expression is reduced and mGluR1-mediated slow EPSCs at parallel fiber synapses are deficient ^78^ ( Table 1). In the conditional SCA1 82Q transgenic mouse line (SCA1 82Q Tre/Tre; tTA/tTA) generated by Zu *et al.* ^79^, stopping the expression of mutant ataxin-1 in Purkinje cells restores mGluR1 expression and pathological phenotypes of Purkinje cells as well as motor dysfunction ( Table 1). In the SCA1 154Q mouse, decreased mGluR1 expression is accompanied by increased expression of mGluR5, which is normally undetectable in adult wild-type mice ^80^ ( Table 1). This is presumably a compensatory effect that rescues Ca ^2+^ signaling to prevent Purkinje cell death. Importantly, enhancement of mGluR1 by a positive allosteric modulator (PAM) improves motor coordination in severely ataxic SCA1 154Q mice ^80^. This result raises the possibility that mGluR1 PAM could be used to ameliorate ataxia in severe SCA1 patients.

**Table 1.** Mouse models of human cerebellar diseases with type-1 metabotropic glutamate receptor (mGluR1) loss of function.

Disease model in mouse	Gene mutation	Changes in expression/function/ localization		Reference
SCA1 82Q	Ataxin-1	mGluR1	Decreased expression Loss of function	Burright *et al.* ^72^ Zu *et al.* ^79^ Shuvaev *et al.* ^81^
SCA1 154Q	Ataxin-1	mGluR1 TRPC3 EAAT4 RORα	Decreased expression	Lin *et al.* ^74^ Skinner *et al.* ^77^ Watase *et al.* ^73^ Serra *et al.* ^75^ Serra *et al.* ^76^
		mGluR5	Increased expression	Notartomaso *et al.* ^80^
*staggerer* mutant	RORα	RORα	Decreased expression	Mitsumura *et al.* ^78^
SCA3	Ataxin-3	mGluR1 RORα	Decreased expression	Konno *et al.* ^82^
SCA5	β-III spectrin	mGluR1	Mislocalization	Armbrust *et al.* ^83^

EAAT4, excitatory amino acid transporter 4; RORα, retinoid-related orphan receptor alpha; TRPC3, short transient receptor potential channel 3.

Very recently, Shuvaev *et al.* ^81^ reported that the SCA1 82Q mouse line (SCA1-Tg; heterozygous B05 line carrying the human Ataxin-1 gene with 82 Q repeats under the control of the Purkinje cell-specific L7 promoter) generated by Burright *et al.* ^72^ exhibited progressive ataxia and impairment in multiple mGluR1 signaling at parallel fiber–Purkinje cell synapses from postnatal week 5 to 12, including TRPC3-mediated slow EPSCs, IP _3_-mediated local Ca ^2+^ signaling in Purkinje cell dendrites, endocannabinoid-mediated short-term synaptic depression, and LTD ^81^ ( Table 1). Importantly, intraperitoneal administration of a GABA _B_R agonist, baclofen, restored mGluR1 signaling at parallel fiber–Purkinje cell synapses and ameliorated ataxia of the SCA1 82Q mouse ^81^. These results are relevant to the *in vitro* studies by Kamikubo *et al.* ^57^ and raise the possibility of a new therapy for SCA1, since baclofen is a clinically available drug ^81^.

Expression of mGluR1 is also reduced in SCA3 and SCA5 mouse models. Konno *et al.* reported that a SCA3 mouse model with disrupted ataxin-3 gene and RORα signaling exhibited impairment of dendritic development and complete loss of mGluR1-dependent endocannabinoid-mediated retrograde suppression of parallel fiber synaptic transmission ^82^ ( Table 1). In a mouse model of SCA5, a mutant form of human β-III spectrin is reported to cause mislocalization of mGluR1 in Purkinje cell dendrites, leading to a functional loss of mGluR1-mediated responses and altered parallel fiber function ^83^ ( Table 1). Taken together, these results suggest that disrupted mGluR1 signaling in Purkinje cells may underlie certain forms of human SCAs.

### mGluR1 gain of function in Purkinje cells and cerebellar ataxia

Recent studies indicate that increased mGluR1 signaling in Purkinje cells could lead to ataxia in several mouse models of human cerebellar dysfunction. Power *et al.* recently reported that the SCA1 82Q mouse line generated by Zu *et al.* ^79^, which is different from the SCA1 Tg-B05 line originally generated by Burright *et al.* ^72^ and used in the recent study by Shuvaev *et al.* ^81^, exhibits reduced motor performance in the rotating rod, reduced complexity of Purkinje cell outer dendrites, decreased height of climbing fiber innervation, and lower frequency of Purkinje cell simple spike firing at 12 weeks of age ^84^ ( Table 2). In contrast to the report by Shuvaev *et al.* ^81^, mGluR1-mediated slow EPSCs and local Ca ^2+^ transients in dendrites induced by repetitive parallel fiber stimulation were both prolonged in SCA1 82Q Purkinje cells without significant changes in their amplitudes ^84^. Remarkably, administration of a negative allosteric modulator (NAM) of mGluR1 shortened the two forms of mGluR1-mediated synaptic responses and ameliorated the ataxia ^84^. These data suggest that mGluR1 gain of function may underlie the pathophysiology of early stage SCA1. However, the data should be interpreted with caution. Power *et al.* reported that blockade of astroglial glutamate transporters, which markedly enhances the amplitude and the duration of mGluR1-mediated slow EPSPs in control mice, had no effect in the SCA1 82Q mouse line ^84^. This result suggests that glutamate uptake by Bergmann glia may be severely impaired in the SCA1 82Q mouse line used by Power *et al.* and, therefore, mGluR1-mediated slow EPSPs may be prolonged, even though mGluR1 signaling itself might be impaired in Purkinje cells similarly to those of the SCA1 Tg-B05 line used by Shuvaev *et al.* ^81^

**Table 2.** Mouse models of human cerebellar diseases with type-1 metabotropic glutamate receptor (mGluR1) gain of function.

Disease model in mouse	Gene mutation	Changes in expression/function		Reference
SCA1 82Q (early stage)	Ataxin-1	mGluR1	Gain of function	Power *et al.* ^84^
SCA2 58Q	Ataxin-2	IP3R	Increased sensitivity	Liu *et al.* ^85^
*moonwalker* mutant	TRPC3	TRPC3	Hyperactive	Becker *et al.* ^87^
SCA14	PKCγ	TRPC3	Hyperactive	Shuvaev *et al.* ^88^

IP _3_R, inositol trisphosphate receptor; PKCγ, protein kinase C gamma; TRPC3, short transient receptor potential channel 3.

In the SCA2 58Q mouse model, mGluR1-induced Ca ^2+^ mobilization through IP _3_R is enhanced in Purkinje cells because of specific binding of mutant ataxin-2 to IP _3_R and elevation of its sensitivity to IP _3_^85^ ( Table 2). Viral delivery of the IP _3_ degradation enzyme IP _3_ phosphatase rescued age-dependent motor incoordination and Purkinje cell loss in the SCA2 58Q mouse model ^86^.

The spontaneous ataxic mutant mouse *moonwalker* exhibits hyperactive mGluR1-mediated TRPC3 currents in Purkinje cells ^87^ ( Table 2). This mouse line has a threonine to alanine switch in TRPC3 that allows the cation channel to open under conditions of weaker mGluR1 activation ^87^. On the other hand, a mouse model of SCA14 has larger mGluR1-mediated inward currents in Purkinje cells than do normal mice because of the failure to inactivate TRPC3 by mutant PKCγ ^88^ ( Table 2). These results suggest that increased Na ^+^ and Ca ^2+^ influx through TRPC3 channels disrupts normal functions of Purkinje cells and other cerebellar neurons, which causes ataxia.

### Dysregulation of mGluR1 signaling in human ataxias

There are several reports supporting the notion that altered mGluR1 signaling in Purkinje cells is related to human cerebellar diseases. Patients who express autoantibodies against mGluR1 ^89^ or Homer-3, a scaffolding protein for mGluRs ^90^, exhibit ataxia. Mutations in mGluR1 ^91^ and TRPC3 have been reported to occur in patients with rare, early onset autosomal-recessive ataxias ^92^. It has been reported that SCA15 is caused by a mutation in the gene encoding the IP _3_R ^93^, whereas SCA14 results from mutations in PKCγ that render this enzyme constitutively active ^94^. These studies suggest that dysregulation of mGluR1 signaling in Purkinje cells may lead to human ataxias.

## Closing remarks

In recent years, several molecules that interact directly with mGluR1 or function downstream of mGluR1 have been identified, and their modes of action have been investigated. These include GluD2 ^70, 71^, TRPC3 ^44, 70^, STIM1 ^69^, GABA _B_R ^56, 57, 95^, A1R ^60, 67^, Ca _V_3.1 T-type Ca ^2+^ channel ^63^, and A-type K ^+^ channel ^68^. It has also become clear that the activation of mGluR1 at parallel fiber–Purkinje cell synapses exerts multiple effects that can induce both elevation and suppression of Purkinje cell activity. These results derive from well-controlled experiments in reduced preparations in which individual phenomena can be isolated either genetically or pharmacologically. It is important to investigate how these multiple effects induced by mGluR1 activation contribute to the net activity of Purkinje cells, development of synaptic wiring onto Purkinje cells, and overall cerebellar function. For this purpose, it would be necessary to examine Purkinje cell responses in intact cerebellum *in vivo* from mice in which a specific mGluR1 signaling molecule has been genetically modified or deleted. *In vivo* whole-cell recording combined with single-cell Ca ^2+^ imaging has been used to record Purkinje cell activity and climbing fiber-mediated responses from intact cerebellum ^96^. It would also be important to record activities from populations of Purkinje cells *in vivo* and analyze their spatiotemporal correlations to investigate the network function of the cerebellum. Genetically encoded calcium indicators and Ca ^2+^ imaging with a two-photon microscope have been used to monitor climbing fiber-mediated responses from populations of Purkinje cells ^97^. On the other hand, since it is not possible to register simple spike activities of Purkinje cells by Ca ^2+^ imaging, conventional extracellular recording is still important.

Another important issue would be the possible diversity of Purkinje cells, other cell types, and neural circuits in different regions of the cerebellum. It has been widely assumed that properties of synaptic transmission, synaptic plasticity, and developmental synaptic refinement, which are mostly based on studies in slice preparations from the cerebellar vermis, apply throughout the cerebellum. However, clear differences exist in gene expression, Purkinje cell firing rates, and behavioral functions of different cerebellar regions ^98^. In this context, Surrathan *et al.* ^99^ recently reported that proper timing between parallel fiber and climbing fiber inputs for LTD induction is different in different regions of the cerebellum such that synaptic plasticity can precisely compensate for behaviorally relevant circuit delays ^99^. Thus, it would be important to investigate whether the same signaling pathways including mGluR1 and its related molecules contribute to LTD in different regions of the cerebellum and how the diversity of synaptic plasticity is produced.

Alteration of mGluR1 signaling has been reported in various clinically relevant mouse models of human cerebellar diseases ^24, 30^. Judging from the severe cerebellar dysfunctions of mGluR1-knockout mice, it is conceivable that mGluR1 loss-of-function underlies human ataxias ^74– 78, 80, 82, 83^. However, it is important to note that mGluR1 gain-of-function has been reported in certain mouse models of human ataxias ^84– 88^. Calcium overload to Purkinje cells due to excess mGluR1-mediated Ca ^2+^ release and/or TRPC3 channel activation is likely to cause cerebellar dysfunction. In such mouse models, down-regulation of mGluR1 signaling molecules may occur to compensate for its hyperactivity with the progress of disease. Thus, it is important to determine whether the mGluR1 loss-of function is the cause or the result of such cerebellar diseases. Given that the recent development of mGluR1 PAM and NAM have raised the possibility of treating cerebellar ataxias, it is important to determine whether mGluR1 signaling is up- or down-regulated at a particular stage of cerebellar ataxia. In addition, a very recent report suggests that baclofen, a clinically available GABA _B_R agonist, can ameliorate cerebellar dysfunction by enhancing mGluR1 signaling in a SCA1 model mouse line ^81^. Careful examination of the mouse models with the progress of cerebellar symptoms would be necessary.
